# Case-based surveillance of antimicrobial resistance in the ACORN (A Clinically Oriented Antimicrobial Resistance Surveillance Network) study

**DOI:** 10.1093/jacamr/dlaa018

**Published:** 2020-03-12

**Authors:** H Rogier van Doorn, Elizabeth A Ashley, Paul Turner

**Affiliations:** 1 Oxford University Clinical Research Unit, Hanoi, Vietnam; 2 Centre for Tropical Medicine and Global Health, Nuffield Department of Medicine, University of Oxford, Oxford, UK; 3 Lao-Oxford-Mahosot Hospital-Wellcome Trust Research Unit, Microbiology Laboratory, Mahosot Hospital, Vientiane, Laos; 4 Cambodia Oxford Medical Research Unit, Angkor Hospital for Children, Siem Reap, Cambodia

Sir,

The authors of a recent opinion piece advocated case-based surveillance of antimicrobial resistance (AMR), including full susceptibility profiles, as opposed to isolate- or sample-based surveillance.^1^ They summarized the three purposes of AMR surveillance as: (i) to inform empiric treatment guidelines and clinical decision-making; (ii) to characterize trends in space and time; and (iii) to provide a benchmark to measure the impact of interventions. A network of systems that fulfil these purposes provides an evidence base to allow for global comparative analyses and to drive strategies for control. The authors summarize the benefits of case-based surveillance as: (i) to allow linking of AMR profiles with patients at risk; (ii) to better inform treatment guidelines; (iii) to identify high-risk populations; (iv) to provide reliable data streams for analyses of effectiveness of interventions; and (v) to study the linkage of AMR phenotypes and burden.

The WHO Global Antimicrobial Resistance Surveillance System (GLASS) provides a platform for standardized data collection and submission and allows for comparison of data between countries and regions. The need for case-based surveillance and its superiority over isolate- or sample-based surveillance, including guidance for implementation, was mentioned in the GLASS guideline for early implementation.^2^ Forty-nine of 69 enrolled countries (as per 31 July 2017) have submitted their data. Most countries submitted isolate- or sample-based data, with low- and middle-income countries (LMICs) relatively underrepresented.^3^ The utility of integrated patient- and laboratory-based surveillance was highlighted in a recent Fleming Fund-funded report on AMR surveillance.^4^ It is expected that large investments, such as the Fleming Fund,^5^ will enhance the capacity of LMICs to conduct surveillance and enrol in GLASS.

Through our work on surveillance in Southeast Asia,^6^^,^^7^ we have identified a number of significant shortcomings of passively collected isolate-based microbiology data. The data collected in our surveillance sites fail to inform clinical decision-making because clinical microbiology services are underused and clinical metadata are lacking. This underuse may be for various reasons, including cost of testing, absence of a ‘culture to culture’ or indeed a microbiology laboratory, slow turnaround time and lack of trust between clinicians and the laboratory. Clinical metadata such as date of hospitalization (including first date of hospitalization for current episode in patients transferred from lower-level hospitals), date of start of antibiotics and clinical syndrome are relevant as they allow stratification of community- and hospital-acquired infection and inform by syndrome. Collectively, the underuse of microbiology and the absence of clinical metadata have in common that they cause overrepresentation of drug-resistant infections among the collected laboratory data; culture will preferentially be performed on more severe and non-responding patients, hospital-acquired infections are generally caused by more resistant pathogens and will contaminate the results if they are not specifically identified, and patients who are already treated with antibiotics when sampled are more likely to grow resistant than susceptible pathogens. The use of such suboptimal surveillance data creates the risk of overestimating resistance and interpretation of these data may lead to advocating broader-spectrum treatment than required and thus may contribute to the problem rather than the solution.

To overcome these issues, we have developed a protocol for clinically oriented AMR surveillance that we are currently piloting in three countries in Southeast Asia (Laos, Cambodia and Vietnam) through partner institutions of the Oxford Tropical Network. The protocol is called ACORN: A Clinically-Oriented Antimicrobial Resistance Surveillance Network. ACORN overcomes most of the issues identified by the authors of the opinion piece, and by us, by adding the following five components to a GLASS-compatible backbone: (i) bedside clinical data collection using a 5 min questionnaire on a tablet, (ii) active case-finding and diagnostic stewardship to support correct use of microbiology diagnostics, (iii) software solutions to link collected data to WHONET-based laboratory data and patient information systems, (iv) 28 day mortality data; and (v) direct feedback of local data using a tailored dashboard. In the pilot phase, ACORN focuses on three clinical syndromes and six pathogens. Patients will be enrolled through daily active case-finding on participating wards using clinical diagnosis/suspicion and weekly prevalence surveys for hospital-acquired infections using case definitions based on those of ECDC.^8^ The protocol for ACORN was submitted for open-access publication and is currently under review.^9^

The current pilot of ACORN will collect data until May 2020, followed by review of the results, identification of challenges and solutions and refinement of the procedures and tools prior to a follow-up proposal for wider roll-out. Expanding the spectrum of clinical syndromes and pathogens will be part of this. Use of case definitions for diagnostic stewardship and enrolment are an area of uncertainty that will be reviewed specifically.

Implementation of ACORN in parallel with laboratory capacity-building in LMICs will maximize the opportunities for rapid generation of actionable AMR and drug-resistant infection data that fulfil the three described purposes of surveillance and will, in addition, provide burden of diseases data.

## Funding

ACORN is funded by Wellcome grant 215867/Z/19/Z to P.T. The funder has not played any decision-making role in writing this letter or its content.

## Transparency declarations

None to declare.

## Supplementary Material

dlaa018_Supplementary_Data
